# Structural Reinforcement Effect of a Flexible Strain Sensor Integrated with Pneumatic Balloon Actuators for Soft Microrobot Fingers

**DOI:** 10.3390/mi12040395

**Published:** 2021-04-02

**Authors:** Satoshi Konishi, Fuminari Mori, Ayano Shimizu, Akiya Hirata

**Affiliations:** 1 Department of Mechanical Engineering, College of Science and Engineering, Ritsumeikan University, Kusatsu 525-8577, Japan; rm0150fs@ed.ritsumei.ac.jp; 2 Graduate Course of Science and Engineering, Ritsumeikan University, Kusatsu 525-8577, Japan; rm0119rs@ed.ritsumei.ac.jp (F.M.); rm0086hx@gmail.com (A.H.); 3 Ritsumeikan Global Innovation Research Organization, Ritsumeikan University, Kusatsu 525-8577, Japan

**Keywords:** strain sensor, pneumatic balloon actuator, inflatable deformation, microchannel, liquid metal, parylene C, integration, soft, flexible

## Abstract

Motion capture of a robot and tactile sensing for a robot require sensors. Strain sensors are used to detect bending deformation of the robot finger and to sense the force from an object. It is important to introduce sensors in effective combination with actuators without affecting the original performance of the robot. We are interested in the improvement of flexible strain sensors integrated into soft microrobot fingers using a pneumatic balloon actuator (PBA). A strain sensor using a microchannel filled with liquid metal was developed for soft PBAs by considering the compatibility of sensors and actuators. Inflatable deformation generated by PBAs, however, was found to affect sensor characteristics. This paper presents structural reinforcement of a liquid metal-based sensor to solve this problem. Parylene C film was deposited into a microchannel to reinforce its structure against the inflatable deformation caused by a PBA. Parylene C deposition into a microchannel suppressed the interference of inflatable deformation. The proposed method enables the effective combination of soft PBAs and a flexible liquid metal strain sensor for use in microrobot fingers.

## 1. Introduction

Generally, robotic systems consist of elemental components including actuators, sensors, and a signal processing controller. Robots can acquire information using sensors and drive actuators based on instructions from a controller. Recently, research on soft robotics using soft actuators and sensors has attracted intense attention, particularly in biomedical applications with a focus on safety and adaptability. Various flexible sensors [1,2,3,4,5] have been developed for wearable sensors, and are also attractive for use in soft robotics. In the application to wearable biosensors, electronic skin, such as biosensing textile-based patch, was reported for sweat monitoring on skin [3]. A wearable patch as a sweat sensor was developed for diabetes monitoring and therapy [4,5]. These wearable sensors employed flexible materials for improved wearability, and their design is likely to be applicable to soft robotics requiring sensors that maintain the original performance of the robot in the presence of bending and other deformations.

Previously, we studied pneumatic balloon actuators (PBAs) for the use in soft microrobots [6,7,8,9,10]. The microrobot finger was designed for a teleoperated tactile sensing feedback robot system. Microfingers were integrated with tactile sensors. A thin film metal thermocouple was developed to detect the temperature at the tip of a microfinger [11]. Strain sensors are required to detect the motion of PBAs for the motion control of soft microrobots. A fluid-resistive sensor was developed as a strain sensor by considering the high compatibility with PBA [12] among the various candidates for flexible sensors [13,14]. PBA uses a fluidic system including a pneumatic balloon structure and microchannels for pressure supply. The design of fluidic channel added as a fluid-resistive sensor enables motion detection of PBAs without requiring an additional special process for sensor integration. Recently, a strain sensor using a microchannel filled with liquid metal was developed for soft PBAs by considering the advantages of sharing common fluidic structures and using electrical signal transmission [15]. Liquid metals such as gallium alloy liquid metals (Galinstan) have been used for wiring and physical sensors [16,17,18].

This study focuses on improving sensor performance with the aim of integrating a flexible strain sensor with soft PBAs, whereas most of previous reports on strain sensors using liquid metals dealt with sensors themselves. The challenges to guarantee performance of devices for integration include interference suppression between sensors and actuators. We noticed the interference by PBAs on integrated sensors in our previous study.

**Figure 1:**
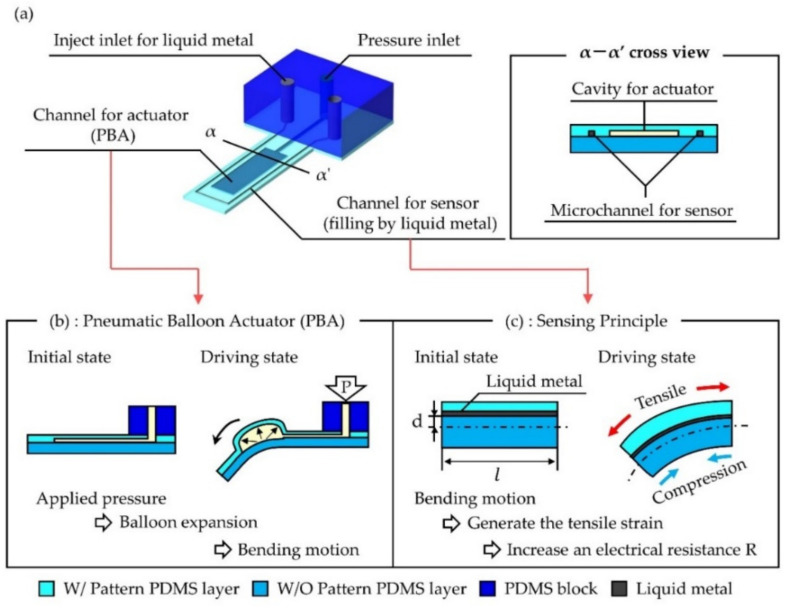
Strain sensor using a microchannel filled with liquid metal for soft pneumatic balloon actuator (PBA). (**a**) Schematic drawing of a single microfinger integrated with the PBA and a liquid metal strain sensor. (**b**) Motion principle of the PBA (side view). (**c**) Sensing principle of uniaxial sensor (side view). (**c**) Triaxial sensor design and its fabrication.

Figure 1a shows a schematic drawing of a single microfinger integrated with the PBA and a liquid metal strain sensor. A cross-sectional view is shown at the right hand. A microchannel and a balloon for PBA and a microchannel for a sensor are formed in the same polydimethylsiloxane (PDMS) structure, so that the common fabrication process can be applied. Liquid metal is filled into the microchannel for a strain sensor. Figure 1b depicts the motion principle of a PBA that moves in a similar manner to a bimorph actuator. PBA is made of PDMS. The bending PBA bends upward due to the strain difference caused by the increase in the internal pressure of the inflatable balloon. Figure 1c illustrates the sensing principle of the liquid metal strain sensor integrated with PBA. The sensor is formed above a neutral axis and the length of the neutral axis is defined as l and the distance between the neutral axis and microchannel for a sensor is d in Figure 1c. The tensile strain generated by the bending motion of the PBA changes the geometry and increases the electrical resistance, R, of the liquid metal sensor because of the positional relation of the neutral axis and the sensor in the case of Figure 1c. The bending motion of a PBA (900 μm × 560 μm × 130 μm) can be detected by the strain sensor using a microchannel (50 μm × 50 μm cross-section) filled with liquid metal. In addition to a uniaxial sensor, a triaxial liquid metal strain sensor was designed and reported for tactile sensing at the tip of a microfinger. Three sensors are aligned every 120 degrees in the circumferential direction. They are bent up and embedded in a PDMS block.

**Figure 2:**
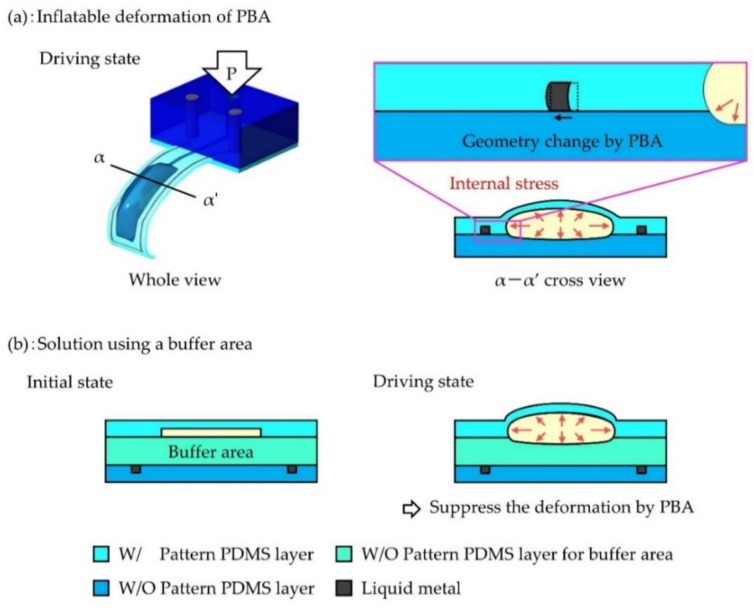
Influence on sensor performance by the inflatable deformation of the PBA and countermeasures for this influence. (**a**) Inflatable deformation of the PBA. (**b**) Solution using a buffer area.

The developed uniaxial liquid metal strain sensor showed linear characteristics for the relative resistance change (ΔR/R_0_) dependence on the bending angle of the PBA. However, the calculated gauge factor (5.89) was much higher than the reference value (1.4) [17,19]. The most likely explanation or the result is that an undesirable geometry change of the sensor occurred due to the internal stress caused by the inflation PBA. A PBA bends due to stress by inflating balloon as shown in the left side of Figure 2a [9]. On the right side of Figure 2a, a cross-section of a microfinger integrated with a PBA and a microchannel for a sensor is shown together with a magnified view. The sensor is deformed due to a stress by inflating balloon in addition to objective bending motion. Therefore, it is necessary to prevent the influence of inflating deformation caused by the PBA. Previously, a buffer layer between the balloon layer and the sensor layer was used in the integrated design of a liquid metal strain sensor into a microfinger using PBAs, as shown in Figure 2b [20]. The buffer layer showed a certain effectiveness for suppressing the influence of the deformation, and the obtained gauge factor was close to the reference value (1.12), where the thickness of the buffer layer was 300 μm. Nevertheless, the influence of inflatable deformation of the PBA still remains, and further improvement is necessary.

This paper describes the integrated design of a liquid metal-based-sensor for the bending motion of PBAs that generates inflatable deformation affecting sensor characteristics by focusing on the geometrical deformation of a microchannel filled with liquid metal. Parylene C film was deposited into a microchannel to reinforce its structure against inflatable deformation caused by the PBA. Parylene C film was employed to reinforce a microchannel structure because of its good surface coverage due to chemical vapor deposition method. It is possible to coat thin parylene C layer on an inner wall of microchannel while keeping original structure of microchannel. It was found that parylene C deposition into a microchannel can suppress the effect of inflatable deformation.

## 2. Materials and Methods

### 2.1. Fabrication Process of a Liquid Metal-Based Sensor for Use with a PBA

**Figure 3:**
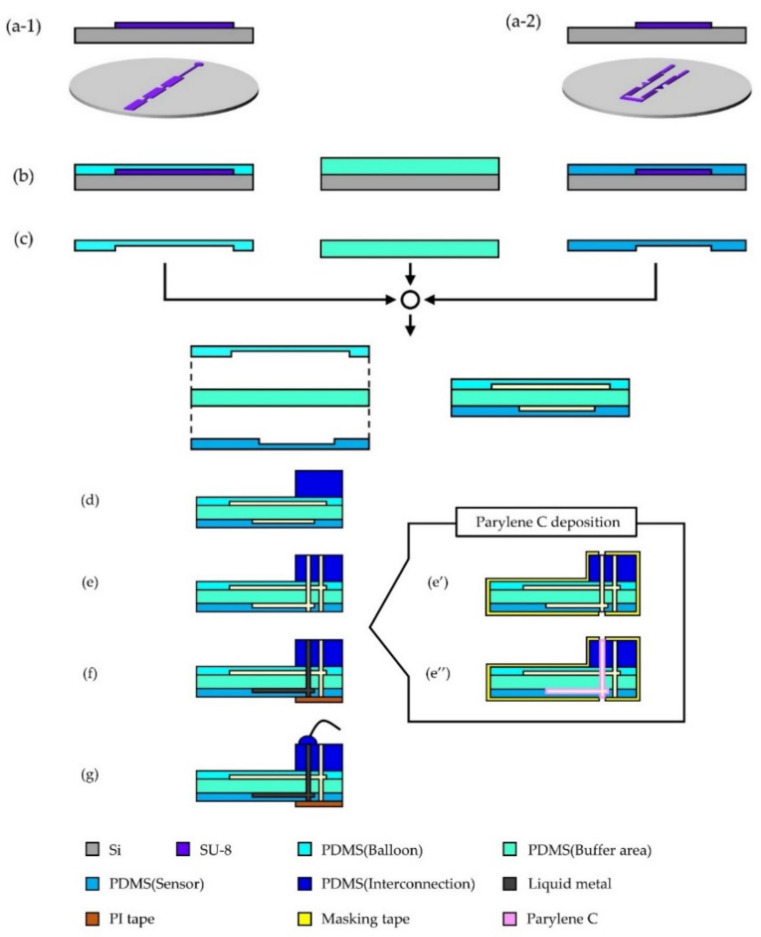
Fabrication process of liquid metal-based-sensor for use with PBAs. Mold structures preparation for PBA (**a-1**) and sensor (**a-2**) on separate substrates. (**b**) Spin-coating formation of three polydimethylsiloxane (PDMS) films for the PBA, a buffer area and a sensor. (**c**) Bonding of three PDMS films by vacuum ultra violet (VUV) technology. (**d**) Implementation of PDMS interconnection block. (**e**) Forming of inlets and microchannels. (**f**) Introducing of liquid metal into microchannels for a sensor. (**g**) Wiring. (**e′**) Masking of structure except an inlet for a microchannel for a sensor. (**e″**) Parylene C deposition into a microchannel for a sensor.

The fabrication process for the integration of a PBA and a liquid metal-based-sensor is illustrated in Figure 3. Both PBA and a strain sensor use microchannel structures formed by molding polydimethylsiloxane (PDMS, Silpot 184, Dow Corning Inc., Bucharest, Romania) on a casting mold. Photoresist (SU-8) mold structures were fabricated on individual Si substrates for the PBA and sensor (Figure 3a1,a2). Figure 3a1,a2 depicts the mold structures for a PBA and the sensor, respectively. PDMS was spun-coated on the mold structures and then cured (Figure 3b). PDMS for the PBA with a 12:1 mixture ratio was spun-coated at 1200 rpm for 40 s and PDMS for sensor with 10:1 of mixture ratio was spun at 800 rpm for 40 s. PDMS for a buffer area was also prepared by spin-coating on a bare substrate. Three PDMS films for a PBA (90 μm thick), a sensor (100 μm thick), and a buffer area (30 μm thick) were bonded together using vacuum ultra violet (VUV) technology as shown in Figure 3c. Then, PDMS interconnection block with inlets and channels was implemented (Figure 3d,e). Galinstan (eutectic gallium indium stannum, Zairyo-ya.com) was injected into microchannels and wired through an interconnection (Figure 3f,g).

As mentioned above, this paper proposes parylene C deposition (Specialty Coating Systems Inc. Working, UK) into the microchannel to suppress the influence of inflatable deformation. Figure 3e′,e″ explains parylene C deposition into microchannel by using masking method prior to a process step of Figure 3f. Approximately 1 μm thick parylene C was deposited in this study. Considering dimensions of the microchannel (50 μm × 50 μm cross-section), the thickness of parylene C was empirically decided in terms of sufficient coverage and structural strength as well as flexibility. We could evaluate the structural reinforcement effect using 1 μm thick parylene C, whereas room remains for further optimization.

### 2.2. Measurement Setup for Sensor Evaluation

The experimental setup for the measurement of the dependence of the electrical resistance of the liquid metal-based-strain sensor on the bending motion of a PBA used a digital multimeter (34,460A, Keysight Tech., Bucharest, Romania) in combination with data processing software (BenchVue). The relative resistance change ΔR/R_0_ was measured and converted into bending angle *θ*.

The deformation of a microchannel for liquid metal-based-sensor with/without parylene C deposition was estimated by applying external pushing force using a positioning stage (KHE06008-C, SURUGA SEIKI CO., LTD., Sanda City, Hyogo, Japan.). The deformation of a microchannel by vertical pushing force was observed by a microscope (VHX 500F, Keyence, Bucharest, Romania). The repulsion force was measured by a load cell (LVS-5GA, Kyowa Electronic Instruments Co., Ltd. Tokyo, Japan).

### 2.3. Estimation of the Gauge Factor

The gauge factor GF and relative resistance change (ΔR/R_0_) of the sensor are related as shown in Equation (1):

(1) $$\frac{\Delta R}{R_{0}}=GF\;\varepsilon ,$$

where *ε* is longitudinal strain of the microchannel.

The strain *ε* is obtained by Equation (2).

(2) $$\varepsilon =\frac{\left(l^{\prime }-l_{0}\right)}{l_{0}},$$

where $l_{0}$ is the length of the neutral axis and $l^{\prime }$ is the length of sensor.

It is assumed that the sensor composed of a microchannel filled with liquid metal is d away from the neutral axis, the strain *ε* is calculated by a central angle θ for arc corresponding to a bending structure as Equation (3).

(3) $$\varepsilon =-\frac{d\theta }{l_{0}}.$$

In this study, the distance d is 145 μm and the neutral axis length $l_{0}$ which is same as the initial length of sensor $l^{\prime }$ is 11 mm. The strain ε and gauge factor GF are calculated using measured resistance R and bending angle θ.

## 3. Results

### 3.1. Fabrication Results of Liquid Metal-Based Strain Sensor Integrated Into Microfinger with PBAs

**Figure 4:**
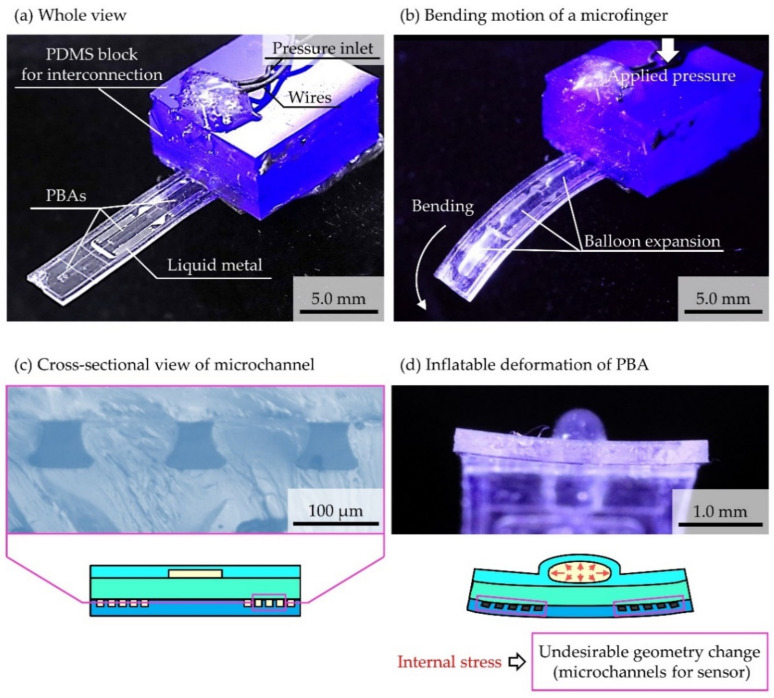
Fabrication results of liquid metal-based sensors integrated into microfinger with PBA. (**a**) Overall view of fabricated microfinger. (**b**) Photograph of bending microfinger. (**c**) Cross-sectional view of a microchannel before filling liquid metal. (**d**) Inflatable deformation of pressurized PBA.

Figure 4 shows the results of the fabrication of microfinger integrated with a liquid metal-based strain sensor and PBA. A developed microfinger was 12.5 mm × 3 mm × 490 μm. An overall view of an integrated device is shown in Figure 4a. The fabricated microfinger contains three PBAs (4 mm × 800 μm × 50 μm) for three-degree-of-freedom. An integrated strain sensor employed a microchannel (50 μm × 50 μm cross-section) filled with liquid metal Galinstan. Figure 4b shows a photograph of the bending motion of the microfinger. A microfinger was bent by 70 degrees when a driving pressure of 100 kPa was applied. Microfinger integrated with flexible liquid metal-based sensor could successfully bend. Figure 4c shows a cross-sectional view of three microchannels before it was filled with liquid metal. Figure 4d shows a view from a tip of microfinger and deformation of pressurized PBA. The microfinger deformed due to the stress generated by the inflatable PBA. We were concerned about an undesirable influence of the inflatable deformation on sensor performance and examined it below.

### 3.2. Influence of Inflatable Deformation by PBA

**Figure 5:**
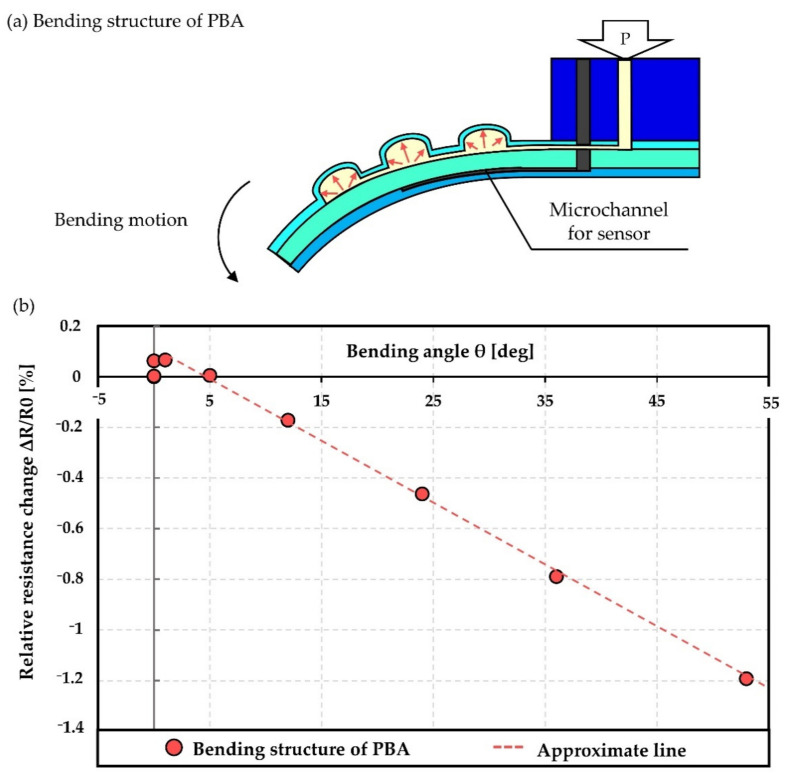
Measured relationship between the bending angle and relative resistance change (ΔR/R_0_) of the sensor. (**a**) Illustration of bending structure of PBA. (**b**) Measured results.

As mentioned above, it is thought that the inflatable deformation of the PBA affects the sensor performance; this problem should be solved to improve the integrated design of the strain sensor into the microfinger using PBA. Figure 5 shows the measured relationship between the bending angle and relative resistance change (ΔR/R_0_) of the sensor. Bending angle of PBA, which is illustrated in Figure 5a, was measured as shown in Figure 5b. A representative result of three measurements is presented in Figure 5. There was an initial rise at a small bending angle due to the dual bending motion of all PDMS PBAs [9]. The bending direction of the microfinger changed from the upper to lower direction at the early stage. The microchannel structure was stressed by the deformation of the PBA when both were arranged close together. A buffer layer acting to prevent a deformation propagation was previously designed to improve the performance. The averaged gauge factor results in approximately 0.89. The sensor was designed to be located at the bottom (ventral of the finger) of the neutrality axis of the finger. The relative resistance change (ΔR/R_0_) decreases due to compressive strain in accordance with the bending angle. On the other hand, the inflatable deformation of the PBA causes tensile strain and the relative resistance change (ΔR/R_0_) increases. The tensile strain caused by the PBA negatively affects sensor performance.

### 3.3. Parylene C Deposition Into Microchannel for Structural Reinforcement of Liquid Metal-Based Sensor

**Figure 6:**
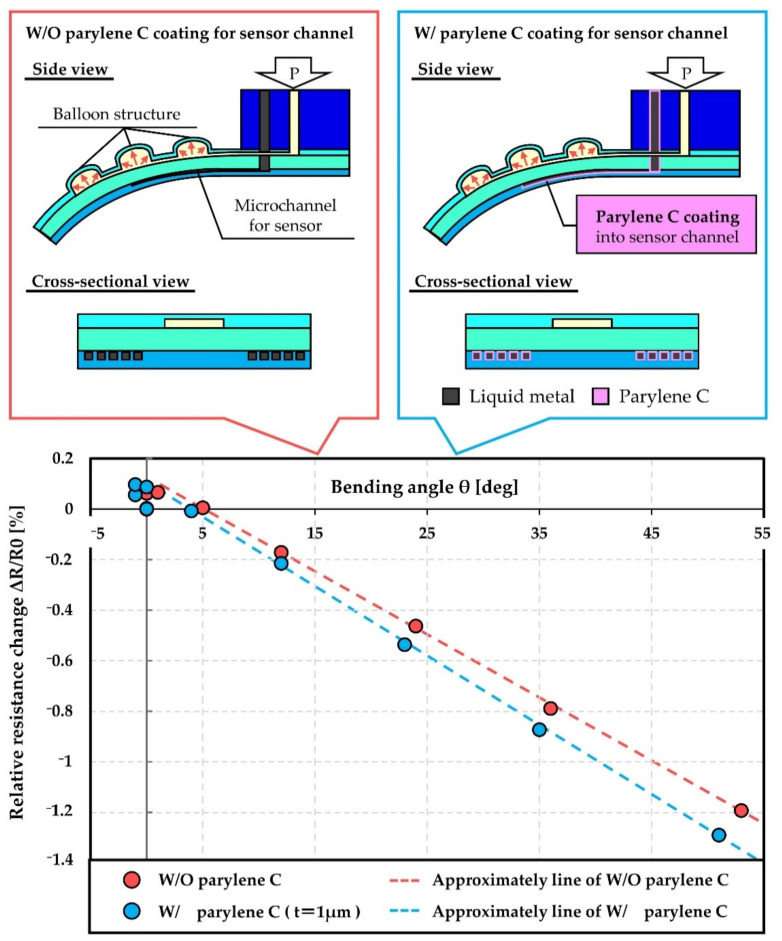
Parylene C deposition in to the microchannel for liquid metal-based sensor and the evaluation of its effect.

In addition to the buffer layer acting to prevent deformation propagation, reinforcement of the microchannel for the sensor structure was designed. Parylene C was selected as the reinforcement material of the inner wall of the PDMS microchannel. Young’s modulus of parylene C and PDMS (10:1 mixture ratio) are reported to be 2.9 GPa [21] and 2.05 MPa [22], respectively. Parylene C is a stiff material compared to PDMS and can be deposited by the CVD process that is suitable for deposition on the inner wall of the microchannel. Figure 3e′,e″ explains parylene C deposition into microchannel. Approximately 1 μm thick parylene C was deposited in this study. Figure 6 shows the results of the evaluation of the reinforcement of the microchannel structure by parylene C deposition. The relative resistance change (ΔR/R_0_) of strain sensors with/without parylene C deposition was compared when they were bent by the PBA. A representative result of three measurements for each condition is presented. The sensitivity of the strain sensor can be improved as a result of the reinforcement of the sensor structure. The averaged gauge factor of the sensors deposited by parylene C was calculated with approximately 1.02, whereas the averaged gauge factor of the sensors without parylene C was 0.89.

## 4. Discussion

The deformation of the inflatable PBA influenced on the characteristics of the strain sensor composed of microchannels filled with liquid metal. Reinforcement of the microchannel structure by parylene C deposition prevented deformation propagation to some extent through the addition to the buffer layer. Further investigation of this effect is discussed below.

### 4.1. Effect of Parylene C Deposition Into Microchannel for Structual Reinforcement

**Figure 7:**
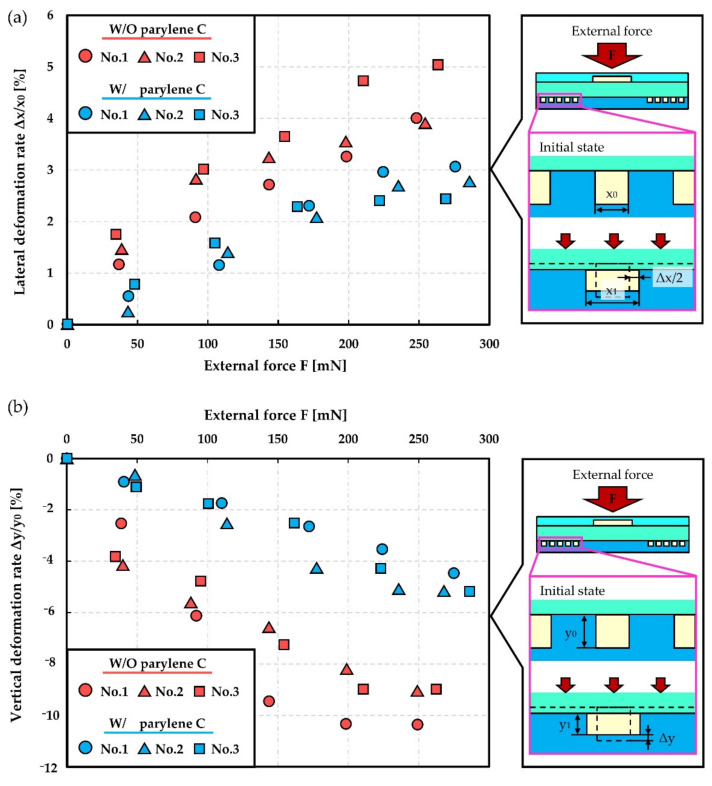

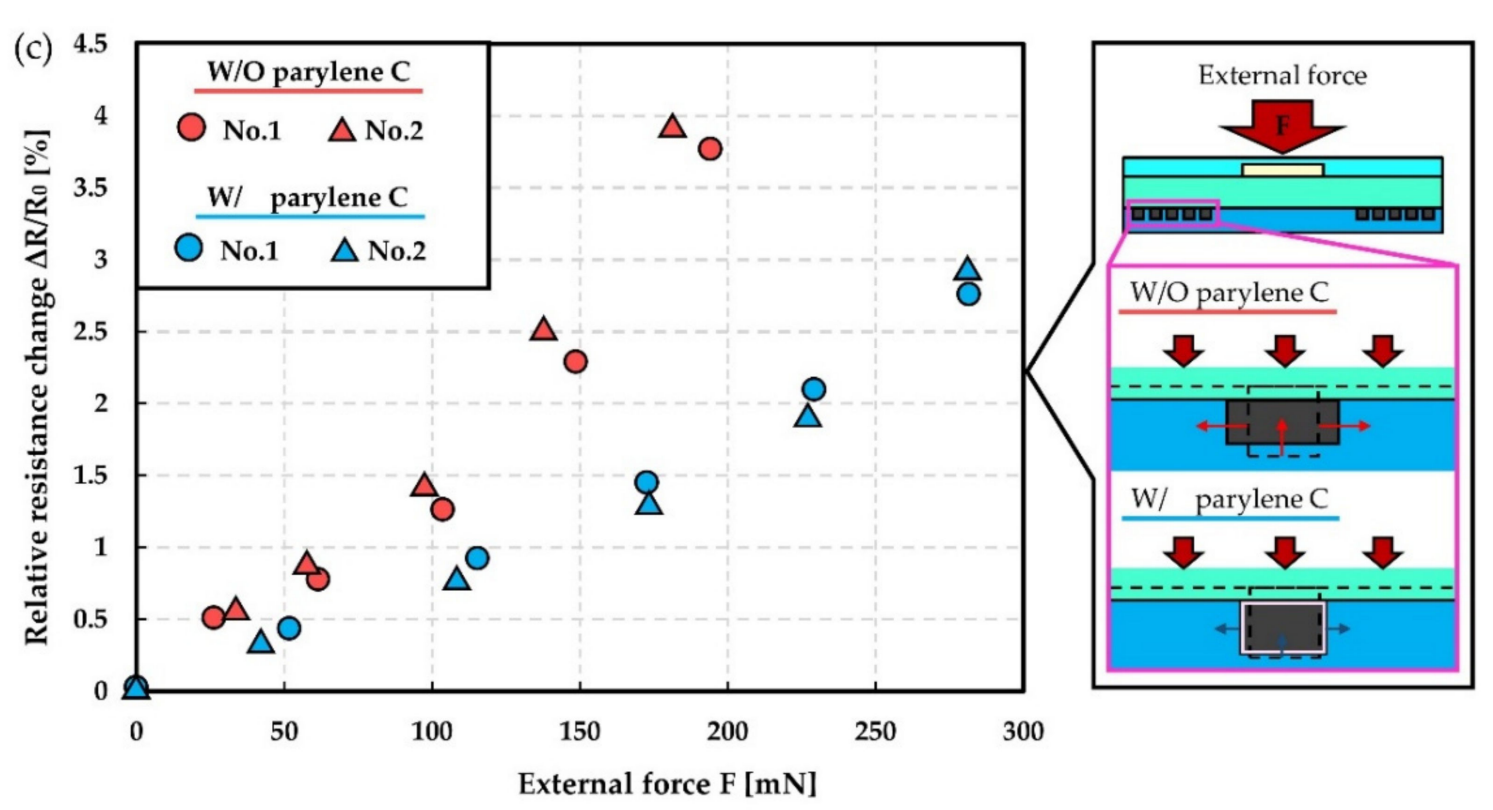
Deformation of the microchannel for liquid metal-based sensor with/without parylene C deposition. (**a**) Vertical geometry change ratio of a cross-section of the microchannel versus external force. (**b**) Lateral geometry change ratio of a cross-section of microchannel versus external force. (**c**) Relative resistance change (ΔR/R_0_) of strain sensors versus external force.

Figure 7 shows the results for the deformation of the microchannel by external force obtained to further understand the mechanism. Figure 7a,b shows the geometry change ratio of a cross-section of the microchannel plotted versus external force provided from the top of the microchannel. Three microchannels with/without parylene C deposition were prepared and compared in the evaluation. The relative resistance change (ΔR/R_0_) of the strain sensors was also measured after introducing liquid metal as shown in Figure 7c. Both vertical and lateral deformation of a microchannel with reinforcement by a parylene C layer were suppressed. The relative resistance change (ΔR/R_0_) of the strain sensor decreased for the microchannel with parylene C. The averaged gauge factor of the strain sensor with/without parylene C deposition resulted in approximately 1.02 and 0.89, respectively. As a result, the gauge factor was improved by approximately 15%. This result can be explained by the effect of the prevention of the propagation of the inflatable deformation caused by the PBA. It is suggested that the effect of reinforcement of the microchannel contributes to the improvement of the gauge factor of the sensor reported in Figure 7.

### 4.2. Effect of Parylene C Deposition into Microchannel for Liquid Metal Introduction

**Figure 8:**
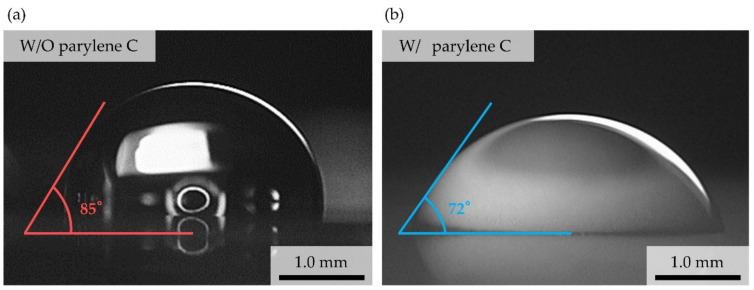
Wettability of the internal wall of the microchannel for liquid metal with (**a**)/without (**b**) parylene C deposition. Water droplet was used in the evaluation.

A microchannel was used to form a flexible strain sensor by introducing liquid metal. It is important to provide a suitable surface as a fluidic microchannel, where parylene C was deposited on the inner wall of a microchannel for the strain sensor in this study. It is not easy to evaluate wettability of Galinstan droplet because of its high surface tension and rapid oxidation in the air [23]. For reference, Figure 8 shows water droplets (20 μL) on the surfaces of PDMS (Figure 8a) and PDMS coated with parylene C (Figure 8b). The contact angles of PDMS and parylene C were 85° and 72° as results of five times tests, respectively. Generally, the improvement of wettability in a microchannel can be expected by parylene C deposition in introducing of liquid metal into a microchannel. The parylene C deposition can be expected to obtain an improvement of wettability for the introduction of liquid metal into a microchannel.

## 5. Conclusions

The integrated design of a strain sensor for PBA using structural reinforcement was presented to suppress the influence caused by the inflatable deformation of the PBA. A flexible strain sensor employed a microchannel (50 μm × 50 μm cross-section) filled with liquid metal in good compatibility with soft PBA. Stiffness reinforcement of a microchannel filled with liquid metal for a strain sensor by deposition of parylene C was proposed in addition to the buffer layer as a deformation propagation preventing structure. The deposition of parylene C into a microchannel suppressed the effect of inflatable deformation. The relative resistance change (ΔR/R_0_) due to the change in the bending angle of the PBA was evaluated. The reinforcement of the microchannel by parylene C showed effect of suppressing of the microchannel deformation and resulted in the improvement of the gauge factor of the sensor. The proposed method can provide an attractive strain sensor suitable for soft PBAs enabling promising applications of soft microrobot systems.

## Data Availability

All data generated or analyzed during this study are included in this published article.
